# Ultrafast Dynamics of Multiple Plexcitons in Colloidal Nanomaterials: The Mediating Action of Plasmon Resonances and Dark States

**DOI:** 10.1021/acs.jpclett.2c01750

**Published:** 2022-07-11

**Authors:** Nicola Peruffo, Fabrizio Mancin, Elisabetta Collini

**Affiliations:** † Department of Chemical Sciences, University of Padova, via Marzolo 1, 35131 Padova, Italy; ‡ Padua Quantum Technologies Research Center, 35122 Padova, Italy

## Abstract

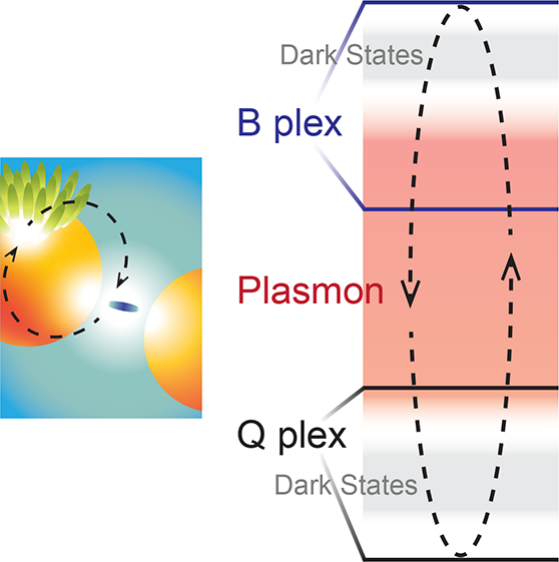

Plexcitons, that is, mixed plasmon-exciton states, are currently gaining broad interest to control the flux of energy at the nanoscale. Several promising properties of plexcitonic materials have already been revealed, but the debate about their ultrafast dynamic properties is still vibrant. Here, pump–probe spectroscopy is used to characterize the ultrafast dynamics of colloidal nanohybrids prepared by coupling gold nanoparticles and porphyrin dyes, where one or two sets of plexcitonic resonances can be selectively activated. We found that these dynamics are strongly affected by the presence of a reservoir of states including plasmon resonances and dark states. The time constants regulating the plexciton relaxations are significantly longer than the typical values found in the literature and can be modulated over more than 1 order of magnitude, opening possible interesting perspectives to modify rates of chemically relevant molecular processes.

During this past decade, the design and preparation of systems capable to enable effective light-matter hybridization have gained increasing interest both in fundamental and applied science.^1,2^ Light–Matter interactions can be activated when an ensemble of quantum emitters (like, e.g., organic molecules and molecular aggregates) is placed in a confined electromagnetic field, generated by optical microcavities or plasmonic structures.^3−6^ A plethora of different phenomena are achievable depending on the coupling strength between the field and the emitters. If resonance conditions are fulfilled and the coupling strength exceeds the mean of their decay rates, the energy levels of the confined field mode and the emitter can be modified; that is, they are *strongly coupled*.^3^ In this regime, two hybrid bands called the upper and lower polariton resonances are formed (UR and LR, respectively), utterly distinct from the initial states of the individual constituent media.^7,8^ The formation of these new resonances is typically identified by the appearance of two split maxima in the absorption spectrum, corresponding to the transition to UR and LR, with an energy gap quantified by the Rabi splitting (ℏΩ_R_).^6^

The knowledge of the mechanisms ruling this coupling and the dynamics of the hybrid electronic states might have a substantial impact on applications in photonics, solar cells, photocatalysis, quantum technologies, and, overall, wherever it becomes relevant to control the energy flux temporally and spatially, even at the nanometric scale.^1,5,9−11^ One of the best ways to reveal how the strong light–matter interaction results in new collective properties is to characterize their ultrafast time-domain behavior.^12−17^ Several studies have been devoted to describing the femtosecond dynamics of several different polaritonic systems, and the debate about the interpretation of the various dynamic phenomena is particularly vibrant. This difficulty is likely due to system-to-system differences, especially in terms of the coupling strength, and a nontrivial dependence on experimental conditions, such as excitation wavelength^18^ and intensity. Therefore, systematic studies of the ultrafast behavior of polaritonic systems and a careful analysis of the dependence on experimental conditions are in high demand to shed light on this debated topic.

To contribute to this effort, here we present a study of the ultrafast dynamics of a family of colloidal plexcitonic nanomaterials. Plexcitons are a specific example of polaritonic states arising from the coupling of the plasmon resonance of a metal nanostructure such as a nanoparticle or a nanostructured metal surface and a molecular excitation.^10,19^ Despite having higher dissipation rates than optical microcavities, metallic nanostructures allow tight confinement of the electromagnetic field of light in sub-wavelength regions, featuring extremely confined mode volumes^20^ and enabling the establishment of effective interactions with other photonic elements in close proximity. Among polaritonic media, the class of colloidal plexcitonic materials is particularly appealing nowadays because these materials are cheap and easy to characterize, and their synthesis is easy to scale up.^8,21,22^

In a previous publication, we characterized the photophysical properties of colloidal multiplexcitonic materials prepared by assembling gold spherical nanoparticles (NPs) and a tetra-sulfonate-phenyl-porphyrin (TPPS) in different aggregation states.^9^ The peculiarity of these systems is that two different sets of resonances can be achieved either in the porphyrin’s B-band region, exploiting the strong coupling of the NPs’ plasmon with the B-band of J-aggregates, or in the Q-band region, forming plasmonic nanogaps with the monomeric species in the intermediate coupling regime.^22^ By acting on the supramolecular interactions regulating the nanohybrid formation, it is possible to build systems where none, one, or both sets of resonances are present. When both plexciton resonances are simultaneously activated in the system, evidence for a plexciton relaxation cascade has been found in static photoluminescence experiments.^9^ Here we employed transient absorption (TA) experiments to clarify the relaxation dynamics of the two sets of plexcitons and assess the presence of possible interactions among them when both resonances are simultaneously present.

The nanosystems under investigation were prepared by assembling 10 nm cationic gold nanoparticles (NPs) and 5,10,15,20-tetrakis-4-sulfonato-phenyl porphyrin in its doubly protonated form (TPPS) in acidic water solutions (pH = 2 in HCl), as described in ref (9). The ratio between NPs and dye concentration governs the supramolecular interactions leading to the nanohybrid formation and the coupling between the plasmonic and the organic moieties.^9^ When the ratio is such to have ∼100 TPPS molecules per NP (sample **1p**, Figure 1), monomeric TPPS molecules cross-link NPs promoting their aggregation and forming the so-called plasmon nanogaps.^23^ In these conditions, the coupling between the porphyrins and the NPs promotes the mixing between the Q-band of TPPS monomer and the plasmon of aggregated NPs, responsible for the red tail of the plasmon resonance, giving rise to two resonances visible in the extinction spectrum at 620 and 670 nm. This was also supported by previous Boundary Element Method (BEM) electrostatic calculations,^9^ suggesting that the plasmon resonance arising from large linear aggregates of NPs can be tuned to be resonant with the monomer Q-band and promote the formation of the UR_Q_/LR_Q_ resonances.

**Figure 1:**
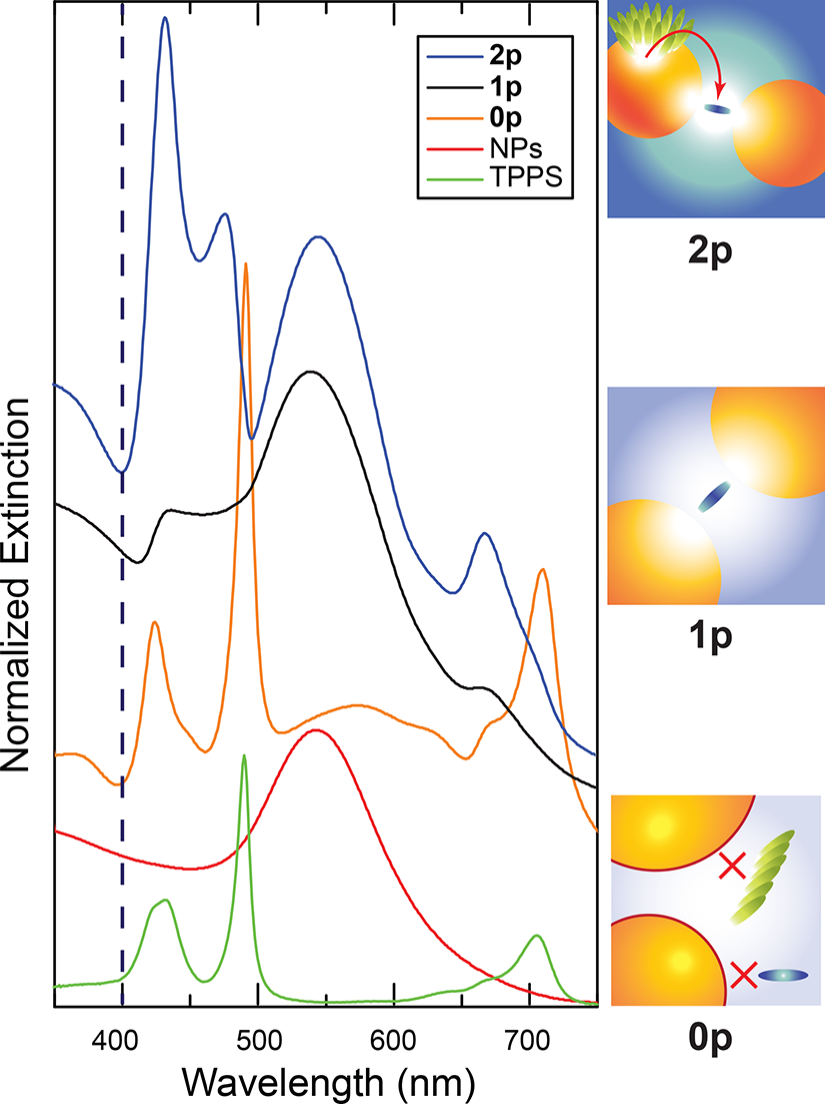
Normalized extinction spectra of TPPS J-aggregates (green), NPs (red), and **0p** (orange), **1p** (black), and **2p** (blue) samples. The spectra were normalized to the plasmon peak in the 500–600 nm spectral region (NPs, **0p**, **1p**, and **2p**) or to their maximum (TPPS) and shifted vertically to ease the comparison. The blue dashed line represents the excitation wavelength (400 nm) used in the TA experiments. A sketch of **0p**, **1p**, and **2p** is reported on the right.

The properties of these resonances, named upper and lower Q resonances (UR_Q_ and LR_Q_), respectively, suggest an intermediate (“Fano”) coupling regime.^9^ At higher amounts of porphyrins in solution (1000 TPPS molecules per NP, sample **2p**, Figure 1), NPs template the formation of J-aggregates. The strong coupling between the aggregates’ B band and the NPs plasmon gives rise to a second set of plexciton resonances, called upper and lower B resonances (UR_B_ and LR_B_). B plexciton states appear in the extinction spectra at ∼475 nm (UR_B_) and 550 nm (LR_B_). B and Q resonances coexist in sample **2p**.

Note that all the supramolecular interactions occur among the cationic capping layer of the NPs and negatively charged sulfonate groups of TPPS. Hence, the addition of a competitor species (i.e., H_2_SO_4_ instead of HCl) allows a drastic reduction of the coupling. The doubly negatively charged sulfate anions, in large excess, inhibit significatively the binding of the porphyrins within the effective volume of the NPs. The extinction spectrum of nanohybrid samples in sulfuric acid (**0p**), Figure 1, except for a slight red shift and broadening of the plasmonic resonance due to NPs aggregation caused by sulfate anions,^9,24^ is basically the superposition of the extinction spectra of the non-interacting species (Figure S1), clear evidence for the absence of relevant coupling.

While a red-shifted and broader plasmon resonance may be associated with greater heterogeneity in the NPs aggregates size and morphology^25^ with respect to the **1p** and **2p**, the **0p** sample together with the noncoupled constituents represents a valuable control to assess the effective role of the coupling in the time-resolved properties of nanohybrids. Indeed, the ultrafast relaxation dynamics of plexcitonic nanohybrids (**1p** and **2p**) has been compared with that of the uncoupled sample (**0p**) and of the building blocks (TPPS and NPs in solutions). Additional details on the procedures used to prepare the different solutions are reported in the Supporting Information.

A TA spectrum plots the differential absorption of the probe beam Δ*A*(*t*, λ) = *A*(*t*, λ) – *A*_0_(λ), with *A*(*t*, λ) and *A*_0_(λ) the absorption spectra with and without the pump excitation, as a function of the probe wavelength λ at a fixed value of the time delay *t* after pump excitation. Instead, the signal decay plots are obtained by plotting Δ*A*(*t*, λ) as a function of the delay time *t* at fixed values of probe wavelengths λ. For all the samples, we recorded Δ*A*(*t*, λ) at different values of pump fluence. More details on the experimental setup used to perform TA measurements can be found in the Supporting Information.

The TA spectra of TPPS in the J-aggregate form at selected values of delay time are reported in Figure 2a. Examples of signal decay at relevant wavelengths are shown in the Supporting Information (Figure S2a). In agreement with previous literature,^26−29^ the TA spectra feature two characteristic strong negative ground-state bleaching (GSB) signals at 490 and 706 nm, in correspondence with the two main excitonic bands. An additional weaker negative signal at 665 nm (vibronic band of the Q transition) and a broad excited-state absorption (ESA) signal between 510 and 650 nm are also recorded. The decays at different probe wavelengths, analyzed with a multiexponential global fit, are described by four main time constants: τ_1_ = 0.6 ps, τ_2_ = 4 ps, τ_3_ = 30 ps, and τ_4_ = 230 ps. The sub-picosecond kinetic (τ_1_) is attributed to the fast relaxation between the B and the Q-band; τ_2_ and τ_3_ are attributed to intra-aggregate and aggregate-solvent energy redistribution, and, finally, τ_4_ is the S_1_ lifetime.^26−29^

**Figure 2:**
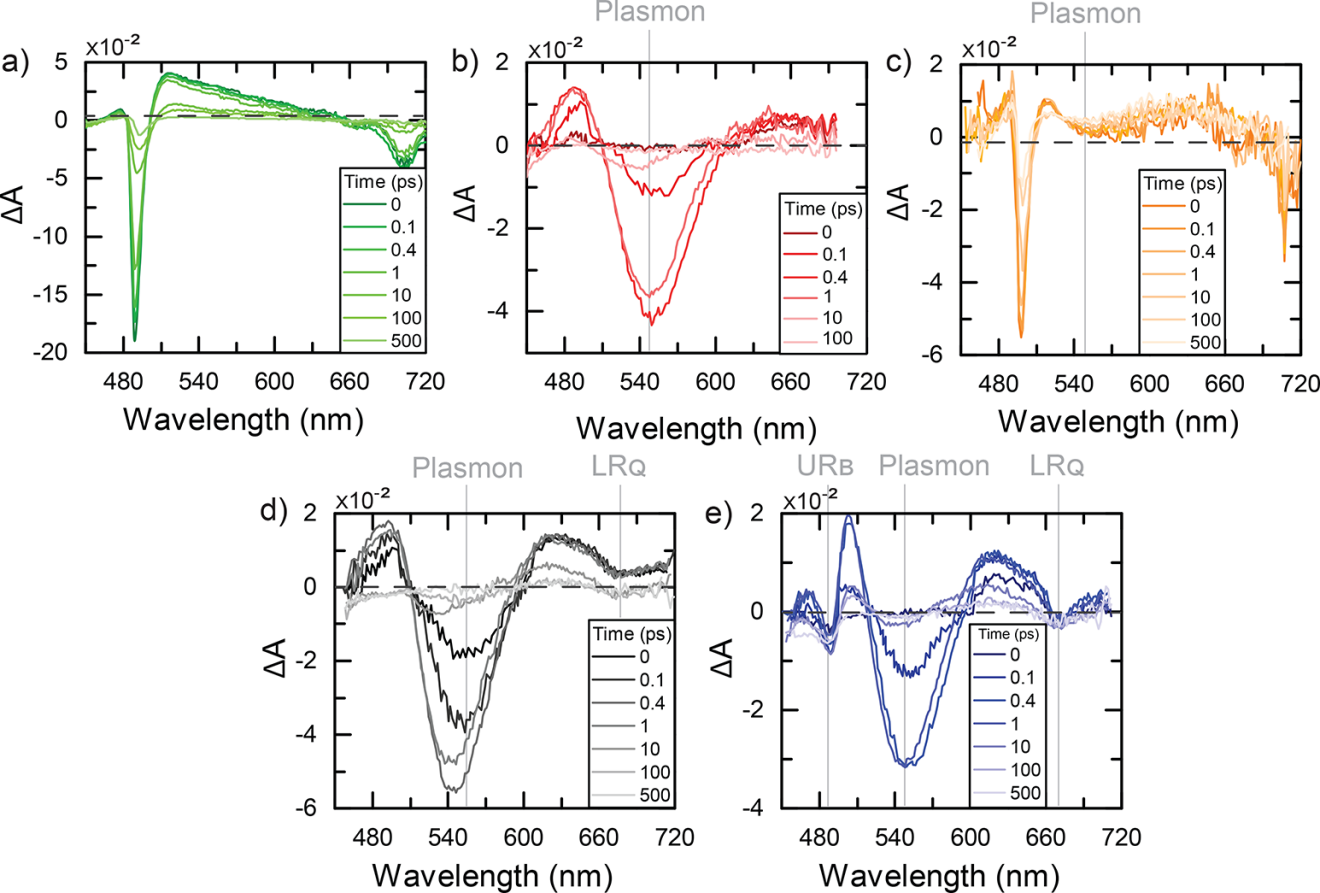
TA spectra at selected delay times for (a) TPPS J-aggregates, (b) NPs sample, (c) **0p** sample, (d) **1p** sample, and (e) **2p** sample, in the same experimental conditions. Delay times are reported in the legends.

The TA spectra of NPs are shown in Figure 2b (the dynamics at selected probe wavelengths are reported in Figure S2b). Also in this case, our findings reproduce previous literature results.^30−32^ A strong GSB signal in correspondence of the plasmon peak dominates the TA spectra. Following the literature,^31^ the relaxation dynamics of NPs have been described with a model taking into account the peculiar nature of the relaxation phenomena in plasmonic nanoparticles: Δ*A*(*t*) = Δ*A*_∞_ + *A*_1_(1 – *e*^–*t*/τ_e–e_^)*e*^–*t*/τ_e–ph_^ + *A*_2_(1 – *e*^–*t*/τ_e–e_^)*e*^–*t*/τ_ph–env_^, where τ_e–e_ is the characteristic electron–electron scattering time constant, τ_e–ph_ is the electron–phonon scattering time constant, and τ_ph–env_ is the phonon-environment time constant. According to that, three kinetic components have been determined: (i) a sub-picosecond rise, attributed to electron–electron scattering (τ_e–e_ = 0.2 ps); (ii) a fast decay associated with electron–phonon scattering (τ_e–ph_, with values between 1 and 3 ps depending on the pump fluence); and (iii) a slower decay due phonon-environment coupling (τ_ph–env_ = 120 ps). All the kinetic constants found in different pump fluence conditions are summarized in the Supporting Information, Table S1. A more detailed comment on the NPs dynamics goes beyond the scope of this work, considering that these data will be used as a comparison for the interpretation of the nanohybrids response. However, it is interesting to highlight that τ_e–ph_ gets shorter as the probe wavelength increases (Figure S2b). This is a typical feature when one probes samples containing NPs’ aggregates.^33^

Figure 2c reports the TA spectra of the **0p** sample at selected times. Qualitatively, the TA spectra roughly appear as the sum of the TPPS and NPs aggregate nonlinearities: two strong negative signals at ∼500 and 700 nm indicate the presence of TPPS J-aggregates, while the broad band at 560 nm is due to the aggregated NPs. However, a closer look reveals that the GSB of the B band of TPPS and the GSB of the NPs plasmon resonance are both slightly red-shifted (from 490 to 500 nm and from 550 to 580 nm, respectively). Although this could be partially justified considering the overlap with ESA signals, it is not unlikely that the redshift is a consequence of the specific organization of the J-aggregates around the gold nanoparticle. Indeed, recent theoretical findings suggest that the three-dimensional (3D) organization of molecules around the surface of a spherical metal nanoparticle in weak coupling conditions leads to the modification of the dipole–dipole interactions and to possible spectral shifts in the optical response.^34,35^ The presence of weak coupling between the nanoparticles and the aggregates in the **0p** sample is supported also by the time behavior of the signals (Figure S2c,d). The dynamics in the 520–570 nm region (Figure S2c) are dominated by the NPs response, although the overlap with the ESA signal of TPPS partially hinders the recognition of all the features and the kinetic constants. In contrast, the dynamics at 500 nm (Figure S2d), in correspondence with the strong GSB signal of the B-band of the TPPS J-aggregates, was fitted with a three-exponential model with time the following constants: τ_1_ = 2.3 ps, τ_2_ = 22 ps, and τ_3_ = 200 ps. With respect to unbound J-aggregates, we recorded an overall shortening of the TPPS kinetics, with the sub-picosecond time constant not even detectable, in agreement with the presence of weak coupling between the aggregates and the NPs.^36,37^

In Figure 2d, the TA spectra at different delay times for **1p** are reported. At wavelengths shorter than 600 nm, the spectra are dominated by the typical broad GSB signal of the NPs, as emerging from the comparison with Figure 2b. In addition, features attributed to the Q Fano resonances are also found. The negative signal at 670 nm is attributed to the GSB of the LR_Q_, whereas no evidence of UR_Q_ was found, probably because of its low extinction coefficient, which makes it barely visible also in the linear extinction spectra.

The same signature of the LR_Q_ GSB at 670 nm was also found in the TA spectra of **2p** (Figure 2e). In addition, another clear negative band at 480 nm is found, attributed to the GSB of UR_B_. By comparison with the response of the NPs and **1p** samples (Figure 2b,d), the strong negative feature centered at ∼550 nm must be attributed also in this case to the dominant contribution of the GSB of the NPs plasmon resonance. However, a further contribution due to the GSB of LR_B_ cannot be entirely excluded.

In addition to negative GSB signals, strong positive ESA signals are recorded at ∼500 and 630 nm. They can be associated with each GSB signal to form a sort of derivative-like band shape, as typically found for polaritonic systems.^14,15,38−42^ Derivative-like features are typically associated with a “contraction” of the Rabi splitting after photoexcitation, which reduces the density of molecules in the ground state able to participate in plexciton formation.^16,43^ In this case, the Rabi splitting is dependent on the pump intensity, and a time-dependent Rabi contraction is measured in TA spectra.^16,41,44^ Since in our experimental conditions we did not observe any significant shift of the plexciton peaks, we attribute these features as originating from the excited-state absorption from the “one-particle” plexcitons and/or the dark states to higher-energy “two-particle” states.^15,16^ Dark states are formed in the nanohybrid as a consequence of the various symmetry of the interactions between a large number of molecules and the plasmon mode, resulting not only in the hybrid plexcitons but also in a large number of purely molecular dark states located at the energy of the bare molecules.^16,45,46^ Dark states get readily populated from higher energy states in time scales less than 100 fs, and, because of their high density, they can efficiently act as a sink of excitation from plexcitons, affecting in a significant way the overall dynamics of nanohybrids, as will be discussed later.^15−17,47,48^ Indeed, energy transfer between polaritons and dark states manifold has been often proposed as a possible mechanism to justify long polariton lifetimes in microcavities.^42,49^

Looking at the time behavior of the signals (Figure 3), in general, for both samples, the time traces present an early time (<10 ps) behavior very similar to the one found for the NPs sample, where a short rise, with a time constant below 1 ps, is followed by a fast decay within 3–10 ps, this latter being fluence-dependent (see Supporting Information, Tables S2–S4). These two dynamics correspond to the already identified electron–electron and electron–phonon scattering processes in NPs. Therefore, we can state that, under the current experimental conditions, the ultrafast dynamics of plexcitonic nanohybrids in the first 10 ps are dominated by phenomena taking place in the plasmonic component. This finding is somewhat expected considering that the experiments have been performed in “off-resonance” excitation conditions, where the pump wavelength, set at 400 nm, predominantly directly excites the sp-to-d bulk interband transition of gold NPs.^32,50^ A similar trend was also recorded in pump–probe experiments on other polaritonic systems.^39^ It was also found that, while τ_e-e_ values do not show significant differences in the nanohybrids and NPs samples, for both **1p** and **2p**, τ_e-ph_ is shorter than in NPs. This difference can be justified by the presence of different anions (H_2_-TPPS^4–^ and sulfate) in the nanohybrid samples, which can influence the nature of the coupling between electrons and phonons and thus slightly change the value of τ_e-ph_. The essential role of the capping layer environment in determining the value of τ_e-ph_ was previously reported also by Shabaninezhad et al.^51^

**Figure 3:**
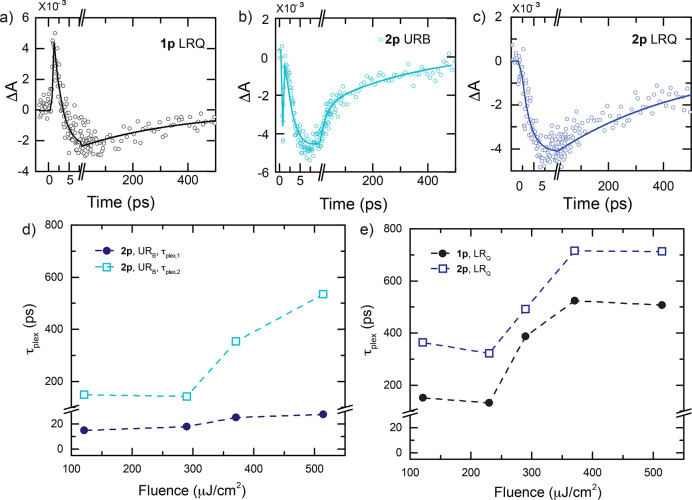
(a) Dynamics of LR_Q_ at 670 nm for the sample **1p**; (b, c) dynamics of the sample **2p** in the UR_B_ region at 480 nm and in the LR_Q_ at 670 nm, respectively. The pump fluence is 290 μJ/cm^2^. The circles represent the experimental data, while the lines are their fittings (note the breaks on the *x*-axes at 10 ps). (d) UR_B_ time constants τ_plex,1,2_ as a function of pump fluence for **2p**. (e) Pump fluence dependence of LR_Q_ time constant for samples **1p** and **2p**.

Instead, the response on a longer time scale (>10 ps) is peculiar to plexcitonic samples and differs from both the bare NPs and TPPS J-aggregate signals. We particularly focus on the dynamics at probe wavelengths 480 and 670 nm, corresponding to the GSB of the identified UR_B_ and LR_Q_ transitions.

Figure 3a–c reports the dynamics of LR_Q_ and UR_B_ for **1p** and **2p** at wavelengths corresponding to the GSB of the associated transitions. Besides the two sub-10 ps (τ_e-e_ and τ_e-ph_) time constants, only one additional time component was necessary to fit the behavior of the LR_Q_ signal (τ_plex_), whereas two components (τ_plex,1_ and τ_plex,2_) were needed for UR_B_ (see also Section S1.2 of the Supporting Information). An inspection of the time constants retrieved from the fitting analysis of the two samples **1p** and **2p** at different values of pump fluence, summarized in Table 1, highlights interesting and somewhat unexpected trends (Figure 3d,e).

**Table 1:** Time Constants Greater than 10 ps Retrieved by the Fitting of Decay Traces at 480 nm (UR_B_) and 670 nm (LR_Q_) for **2p** and **1p** Samples at Different Pump Fluences (in μJ/cm^2^)^a^

	**2p**			**1p**
	UR_B_		LR_Q_	LR_Q_
fluence	τ_plex,1_	τ_plex,2_	τ_plex_	τ_plex_
514	28	534	712	507
371	25	353	715	524
290	18	142	493	388
121	15	149	363	151

a The fitting function, the sub-10 ps time constants (τ_e-e_ and τ_e-ph_), and the amplitudes of the different exponential terms are reported in the Supporting Information (Section S1.2 and Tables S2–S4). The error is estimated in the order of 10% from repeated measurements.

First, these time constants are far longer than the average lifetimes typically reported for other plexcitonic and polaritonic systems, including Fano resonances, typically in the order of 0.1–10 ps.^14,38,39,41,52−55^ The long-living dynamics is particularly surprising for UR_B_ because several works reported sub-100 fs relaxation dynamics for upper branches of polaritons (often not even resolved in pump–probe experiments) due to the ultrafast downhill relaxation to dark states or to the lower polariton branch.^43,44^ Moreover, the time constants manifest a marked fluence-dependent trend in all cases, with values increasing at higher fluences.

Second, the temporal trace of the transient signal at 670 nm (describing the recovery of the GSB of LR_Q_) is significantly different in **2p** and **1p**. In the **2p** sample, when the B plexciton is also present, the recovery of the LR_Q_ bleaching doubles its time constant.

The comparison between the dynamic trends for the **1p** and the **2p** samples is particularly interesting, especially from what concerns possible relaxation processes between UR_B_ and LR_Q_ in **2p**. Comparing Figure 3b,c, we did not find in **2p** evidence for kinetic components depicting a decay of UR_B_ and a concomitant rise of LR_Q_ signal, suggesting the absence of direct downward relaxation pathways among the two sets of resonances, at least in the considered experimental conditions and in the investigated time window. This finding adds to previous static fluorescence measurements, where emission from the Q plexciton could be recorded only upon excitation of the B plexciton,^9^ and supports the hypothesis that the photophysical and dynamical properties of the two sets of resonances in **2p** are strongly intertwined. Rather than considering the B and the Q resonances as two separated sets of states connected by an incoherent downhill energy transfer, more subtle interactions should be considered, possibly mediated by the plasmonic moiety and dark states, as discussed below and sketched in Figure 4.

**Figure 4:**
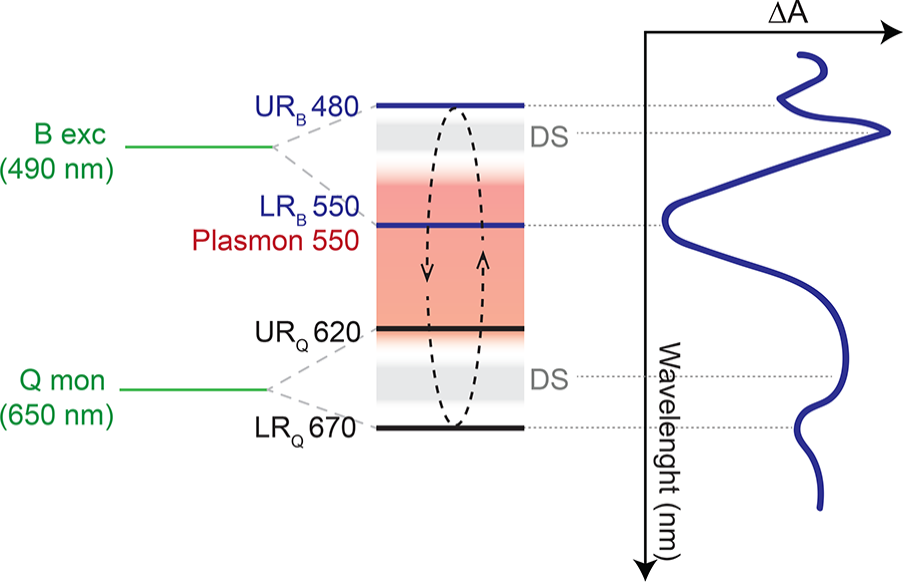
Energy-level scheme for sample **2p** (left) compared with the measured TA spectrum (right). Dark states (DS) and plasmon resonances are represented in gray and red, respectively. The numbers indicate the corresponding wavelengths. In green, we report the molecular states giving rise to the B and Q plexcitons manifolds (the B exciton of the TPPS J-aggregate at 490 nm and the Q-band of the monomeric TPPS at 650 nm). The dashed black arrows illustrate the population redistribution mechanism between UR_B_/LR_B_ and UR_Q_/LR_Q_ mediated by plasmon resonances and dark states.

Generally, the common intuition is that population dynamics flow spontaneously downhill. However, recent theoretical and experimental papers are challenging the intuitive connection between the spectroscopic ordering of states and the spontaneity of the energy flow in strongly coupled polariton states. This is mainly due to the specific nature of the interactions between polaritonic resonances and what can be generally defined as the “continuum reservoir”. Indeed, the energy landscape in between UR_B_/LR_B_ and UR_Q_/LR_Q_ presents a high density of other states potentially involved in the relaxation dynamics, including: (i) the reservoir of states belonging to uncoupled molecules, both in the form of J-aggregates and monomers, (ii) plasmon resonances, and (iii) the manifold of dark states formed upon plasmon-exciton hybridization.

A significant contribution by uncoupled molecules (i) can be ruled out, considering that the TA spectra and the dynamics of the nanohybrids are noticeably different from the ones of the uncoupled molecular species. This is also supported by the comparison with the response of sample **0p**.

As already discussed, the heavy involvement of NPs plasmon resonances (ii) is instead witnessed by the presence of the strong GSB signal of the plasmonic resonance at ∼550 nm that dominates the TA spectra of the nanohybrids. Moreover, the early time dynamics (<10 ps) captured for both **1p** and **2p** in UR_B_ and LR_Q_ regions strongly resemble that of bare NPs, with similar fluence and wavelength dependence. It is reasonable to state that the initial excitation promoted by the ultrafast pump pulses directly into the NPs interband transition at 400 nm is transformed into a nonequilibrium distribution of electrons and holes in the metal, whose relaxation then strongly affects the overall nanohybrids dynamics because metallic excitations can directly exchange energy with the adsorbed molecules. Our data suggest that the continuum of states of the plasmon band must necessarily be considered for an accurate description of the observed dynamic behavior. Interestingly, a similar hypothesis was formulated also to explain why the sub-picosecond dynamics of an Ag NPs-cyanine J-aggregate system was dominated by the electron–electron scattering time constant of the NPs moiety.^17^

The substantial contribution of the plasmon band to the overall nanohybrids dynamics explains why the LR_Q_ lifetime slows down at higher fluence and in the presence of the B plexciton. Indeed, the interband excitation of gold NPs at 400 nm produces conduction-band electrons. The excess energy is then released mainly to the lattice.^30,32,56^ As the “heating” of the lattice becomes stronger, at increasing fluence and at higher photon energies, the thermalization process slows down,^50^ according to the well-known two-temperature model.^57,58^ The fluence-dependent behavior of the nanohybrids depicted in Figure 3d,e has likely the same origin.

This interpretation is also supported by previous photoluminescence measurements that reported the quenching of the emission from LR_Q_ when the temperature was lowered to 77 K.^9^ This evidence implies that the mechanism of population of the emitting state is annihilated at low temperature.

In addition to plasmons, dark states (iii) must also be taken into account. The role of dark states has already been widely discussed to rationalize the transient femtosecond behavior of polaritonic media, and it has been recently reviewed in ref (16), where an interesting view based on free energy considerations is proposed. It has been suggested that the high content of entropy of the dark states can give rise to a quasi-equilibrium between the lower branch of the polariton resonance and dark states manifold, providing a working mechanism for uphill population transfer from the lower polariton to higher energy states. This assumption allows one to justify peculiar experimental observations such as unexpectedly long dynamics of the UR^15^ and anti-Stokes fluorescence.^59^ A similar concept was also proposed in ref (60), where the incoherent exchange of energy between polaritons and reservoir states was postulated to alter the population damping of the hybrid states, strongly affecting the polariton dynamics. It was predicted that this energy exchange could repopulate (depopulate) the upper (lower) polariton, justifying the microscopic origin of the sub- or super-radiance of the hybrid modes. The presence of an exchange of energy between dark states and polariton resonances has also been invoked to explain polariton lifetimes longer than expected.^16,42^

In our samples, the presence of dark states directly involved in the dynamics is manifested through the appearance of ESA signals in between the GSB bands of the plexciton resonances (Figure 2d,e). These states could be effectively involved in redistributing the population not only among the lower and upper branches of the same plexciton resonance but also among the B and Q branches. Moreover, the population redistribution mediated by the dark states is expected to be enhanced in our samples by the contribution of metal states. Indeed, as discussed before, the nonequilibrium distribution of electrons and holes promoted in the NP moiety by the 400 nm excitation is expected to generate a rise in the lattice temperature. This temperature rise increases the entropic contribution of dark states, which favors the establishment of a quasi-equilibrium between the plexciton branches and dark states manifold and promotes uphill population transfer.^16^ This agrees with recent MD simulations that confirmed that a redistribution of the thermal energy could indeed boost the occupation of higher-energy polaritonic states.^61^

The synergic action of a plasmon band and dark states in promoting a fluence-dependent population redistribution among the different branches of the two resonances, also through uphill energy transfer, can explain the intertwined dynamics of UR_B_ and LR_Q_, the absence of the expected direct downhill energy transfer from B to Q manifolds, and the fluence-dependent lifetime extension of LR_Q_. This mechanism was made possible thanks to the peculiar energy position of dark states and plasmon resonances with respect to Q and B plexciton resonances, as depicted in Figure 4, and by the specific excitation at 400 nm.

Even more interesting, such a mechanism is expected to be active only for plexciton systems built on colloidal nanoparticles, where the molecules are in close contact with the NP surface guaranteeing an efficient exchange of energy among plasmon resonances of the NP and the dark states localized on the molecular counterpart. It is likely that the same mechanism cannot be significantly activated in microcavity polaritons, where the molecular layer is typically separated from the metal mirrors by isolating layers, and it lays at the antinode of the cavity.^62^ This would explain the different behavior of other polaritonic materials based on TPPS J-aggregates under 400 nm excitation.^63,64^

Overall, our data confirmed that the ultrafast dynamics of plexcitonic nanohybrids are strongly affected by the presence of a reservoir of states including plasmon resonances and dark states and can be tuned acting on the experimental conditions (excitation wavelength and fluence). By considering samples with a different number of interacting plexciton resonances (so to change the distribution of states belonging to the “reservoir”) and modulating the pump fluence, it was possible to record relaxation dynamics with time constants spanning a range of at least 1 order of magnitude.

While further investigations on different nanohybrids under different experimental conditions (especially excitation pump wavelength) are still needed to verify the generality of this phenomenon, our findings suggest that it should be possible to exploit these mechanisms to control the dynamics of plexciton systems over a significantly large time span. This opens a possible interesting perspective for effectively using the strong coupling to modify rates of molecular processes and chemically relevant reactions.

## Supporting Information Available

Additional details about samples preparation and transient absorption setup; additional data sets (UV–vis spectra, TA data and fitting parameters tables) (PDF)
