# Supplementary material for: Ultrafast Dynamics of Multiple Plexcitons in Colloidal Nanomaterials: The Mediating Action of Plasmon Resonances and Dark States
Source: J Phys Chem Lett. 2022 Jul 11;13(28):6412–9. doi: 10.1021/acs.jpclett.2c01750 (PMC9310092; doi:10.1021/acs.jpclett.2c01750)
Supplement: Supplementary file 1 — jz2c01750_si_001.pdf [file jz2c01750_si_001.pdf]

SUPPORTING INFORMATION

Ultrafast Dynamics of Multiple Plexcitons in Colloidal Nanomaterials: The Mediating Action of Plasmon Resonances and Dark States

Nicola Peruffo,[1] Fabrizio Mancin[1], Elisabetta Collini,[1,2]*

*elisabetta.collini@unipd.it

[1] Department of Chemical Sciences, University of Padova, via Marzolo 1, 35131 Padova, Italy.
[2] Padua Quantum Technologies Research Center

Contents

S1. Experimental methods
S.1.1 Sample preparation
S1.2 *Transient absorption (TA) measurements.*

S2. Additional data
S2.1 Additional UV-Vis spectra
S2.2 Additional TA data and Tables

S1. Experimental methods

S1.1 Samples preparation

Citrate capped gold nanoparticles were prepared according to a modified version of a literature procedure.[1] Typically, 1.65 mL of sodium citrate (510 mM), 250 μL of silver nitrate (10 mM) and 500 μL of tetrachloroauric acid (253 mM) were added in this precise sequence to 5.6 mL of water, under vigorous stirring. The resulting solution is stirred for 5 min. During this time, the solution changed from the initial yellow color to green. After the incubation time, the solution was quickly added to 117 mL of boiling water and heated under reflux for 1 h, becoming wine-red after a few seconds. The resulting citrate-capped nanoparticles solution was then allowed to cool down to room temperature. The 8-trimethylammonium octylthiol capping molecule was synthesized following the procedure described in ref. ([2]). 2 mL of the 8-trimethylammonium octylthiol solution (33mM) were added to the solution of nanoparticles, and the mixture was allowed to react overnight under stirring. The solution was filtered by a 0.2 μm syringe filter to remove large aggregates and then, to eliminate the citrate impurities, it was washed five times with a 100kDa cutoff filter (Amicon® - Ultra) (previously washed three times with 1:1 EtOH:water).

In our previous work,[3] we characterized the average diameter of these 8-trimethylammonium octylthiol-capped nanoparticles (NPs) being 11 ± 2 nm, and their averaged formula $Au_{30891}(SR)_{2426}$ (where SR is the cationic thiol). According to this formula, for convenience, the NPs concentration has been converted into concentration of 8-trimethylammonium octylthiol units grafted on their surface by multiplying by 2426. The NPs are well dispersed in solution, with a plasmon resonance centered at 520 nm (Figure S1).

0p was prepared as follows: the pH of a 1M solution of Na_2SO_4 was adjusted to 2 with H_2SO_4; successively 10 μL of a 1 mM Na-TPPS solution (pH=7) were added to 480 μL of the latter solution. Successively, 10 μL of a 5 mM solution of nanoparticles above synthesized were added. **1p** and **2p** were prepared by adding 5 μL of TPPS (pH=3) to a water solution (pH=2) and successively adding 25/5 μL of a 5 mM solution of nanoparticles above synthesized.

In hybrid TPPS-NPs samples (**0p**, **1p**, and **2p**), nanoparticles are aggregated, as attested by the redshift of the plasmon resonance to the 550-580 nm region. For this reason, we used as a reference a sample containing aggregated NPs. This sample was prepared simply by adjusting the pH of the solution of nanoparticles to 2 with H_2SO_4. The sulphonate anions strongly crosslink the nanoparticles and induce aggregation. The plasmon resonance of the aggregated nanoparticles in the NPs sample is thus shifted from 520 nm to 550 nm (see figure 1).

S1.2 Transient absorption (TA) measurements.

TA measurements were performed using a home-built pump-probe setup. The source is an amplified Ti:Sapphire laser (Mai-Tai+Spitfire, Spectra Physics) that emits pulses at 800 nm with energy of 0.8 mJ per pulse, repetition rate of 1 kHz, and 160 fs pulse duration. The output laser beam is split by a 4% beam splitter into two paths. The weaker one generates a super-continuum white light in a thin sapphire plate and is used as the probe. The second more intense portion is used to obtain the pump pulse at 400 nm via second harmonic generation in a BBO thin crystal. The pump fluence is tuned from 121 to 530 μJ/cm^2 using OD filters and its repetition rate is halved to 500Hz through an optical chopper. The collimated pump pulse and the focused probe pulse are then spatially overlapped in the sample, hold in a 1mm quartz cuvette. In the overlap position, the pump and the probe beams have a diameter of about 70 μm and 25 μm, respectively. The delay between pump and probe pulses is controlled with a motorized linear stage. The transmitted light is dispersed and directed to a linear CMOS diode array detector.

A TA spectrum plots the differential absorption $\Delta A(t,\lambda)$ as a function of the probe wavelength λ at a fixed value of the time delay t after pump excitation. $\Delta A(t,\lambda) \propto -log\ (I(t,\lambda) - I_0(\lambda))/I_0(\lambda)$, where $I_0(\lambda)$ and $I(t,\lambda)$ are the intensity of the signal at probe wavelength l without pump excitation and at a time delay t after pump excitation, respectively. Negative ΔA signals are the result of stimulated emission (SE) and ground-state bleaching (GSB) and correspond to the creation of excited state population at the one-particle states. Positive ΔA signals are instead the result of excited state absorption (ESA) from the one-particle to the two-particle states. The quality of the TA signal is improved through repeated measurements and averaging (150-200 measurements were averaged to obtain a sufficient signal-to-noise ratio). The obtained spectra are numerically processed to minimize white light chirping effects, by using a home-made Matlab routine. Each measure is repeated at least twice to verify the reproducibility of the phenomena.

The collected data can be shown in terms of TA spectra (ΔA vs λ) at fixed values of delay time t, as in Figure 2 of the main text, or in terms of decays (ΔA vs t) at fixed values of probe wavelengths λ, as in Figure 3a-c.

In the analysis of the decay traces, different fitting models have been considered for different samples to account for the diverse nature of the involved states and dynamic processes. For the uncoupled molecular samples, the typical multiexponential fitting model was employed: $\Delta A(t) = \Delta A_\infty + \sum_n A_n e^{-t/\tau_n}$, with ΔA_∞ the background value at longer times, τ_i the time constants and A_i their corresponding amplitudes. For the uncoupled nanoparticles samples, a more complex model has been used to take into account the different nature of the relaxation phenomena: [4]

$$\Delta A(t) = \Delta A_\infty + A_1\left(1 - e^{-\frac{t}{\tau_{e-e}}}\right)e^{-t/\tau_{e-ph}} + A_2\left(1 - e^{-\frac{t}{\tau_{e-e}}}\right)e^{-t/\tau_{ph-env}} \quad \text{Eq S1}$$

where $\tau_{e\text{-}e}$ is the characteristic electron-electron scattering time constant, $\tau_{e\text{-}ph}$ the electron-phonon scattering time constant and $\tau_{ph\text{-}env}$ the phonon-environment time constant.

Finally, for the study of the nanohybrids the following function has been used to take into account that the dynamics <10 ps basically coincide with the dynamics of NPs:

$$\Delta A(t) = \Delta A_\infty + A_1\left(1 - e^{-\frac{t-\tau_0}{\tau_{e-e}}}\right)e^{-t/\tau_{e-ph}} + \sum_n A_n e^{-t/\tau_{\text{plex,n}}} \quad \text{Eq S2}$$

Only one additional time ($\text{n} = 1, \tau_{\text{plex}}$) was needed to fit the LR_Q GSB recovery at 670 nm, while two time constants ($\text{n} = 2, \tau_{\text{plex,1}}$ and $\tau_{\text{plex,2}}$) were needed to fit the behavior at 480 nm for the GSB of UR_B.

S2. Additional data

S2.1 Additional UV-Vis spectra

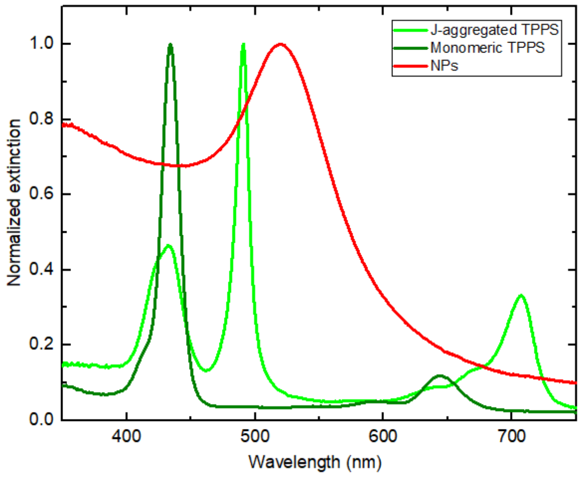

Figure S1. Normalized linear extinction spectra of uncoupled components: not aggregated NPs and the monomeric and aggregated form of TPPS.

S2.2 Additional TA data and tables

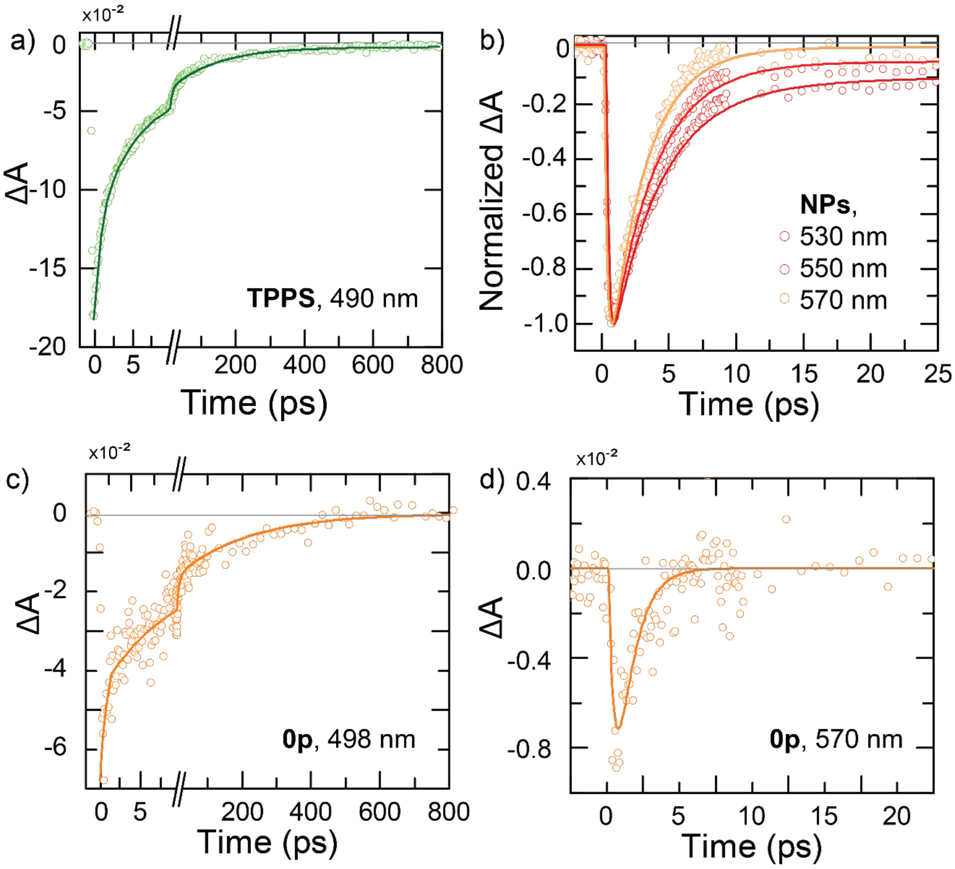

Figure S2. Decay traces at selected probe wavelengths for: (a) TPPS J-aggregates, (b) NPs, (c-d) **0p**. The probe wavelengths are reported in the legends. In panel (b), each track is normalized to the minimum to ease the comparison. The pump fluence is 330 μJ/cm^2.

Table S1. Fitting results (Eq S1) of NPs at selected probe wavelengths and for different values of pump fluence. The error is estimated in the order of 10% from repeated measurements. Fitting amplitudes are reported between square brackets.

Pump fluence ($\mu J/cm^2$)	Probe wavelength (nm)	τ_{e-e} (ps)	τ_{e-ph} (ps) [A_1]	τ_{ph-env} (ps) [A_2]
330	480	0.22	3.7 [0.015]	
	520	0.18	3.2 [-0.016]	80 [-0.0045]
	530	0.18	3.7 [-0.035]	130 [-0.0044]
	540	0.20	3.6 [-0.049]	180 [-0.0041]
	550	0.20	3.3 [-0.057]	410 [-0.0029]
	560	0.20	3.0 [-0.056]	
	570	0.23	2.7 [-0.047]	
	580	0.18	2.2 [-0.036]	
	670	0.22	2.3 [0.0046]	
110	480	0.22	2.4 [0.0089]	
	520	0.28	2.7 [-0.12]	160 [-0.0016]
	530	0.29	2.6 [-0.024]	140 [-0.0021]
	540	0.25	2.5 [-0.032]	310 [-0.0016]
	550	0.25	2.2 [-0.034]	
	560	0.18	2.1 [-0.030]	
	570	0.21	1.7 [-0.023]	
	580	0.17	1.3 [-0.015]	
	670	0.21	1.7 [0.0027]	

Table S2. Fitting results (Eq S2) of **2p** at the probe wavelengths corresponding to the GSB of UR_B (480 nm) for different values of pump fluence. The error is estimated in the order of 10% from repeated measurements. Fitting amplitudes are reported between square brackets.

UR_B (480 nm)				
Pump fluence ($\mu J/cm^2$)	τ_{e-e} (ps)	τ_{e-ph} (ps) [A_1]	$\tau_{plex,1}$ (ps) [A_2]	$\tau_{plex,2}$ (ps) [A_3]
514	0.5	2.3 [0.016]	30 [-0.0026]	530 [-0.0061]
371	0.6	1.7 [0.026]	25 [-0.0018]	350 [-0.0042]
290	0.5	2.1 [0.012]	20 [-0.0024]	140 [-0.0027]
121	0.3	1.4 [0.0078]	15 [-0.00087]	150 [-0.0014]

Table S3. Fitting results of **1p** and **2p** (Eq S2) at the probe wavelengths corresponding to the NPs plasmon resonance (520-580 nm) for different values of pump fluence. The error is estimated in the order of 10% from repeated measurements. Fitting amplitudes are reported between square brackets.

Pump fl. ($\mu J/cm^2$)	Probe wav. (nm)	τ_{e-e} (ps)		τ_{e-ph} (ps) [A_1]		τ_{plex} (ps) [A_2]	
		1p	2p	1p	2p	1p	2p
514	520	0.22	0.29	2.9 [-0.078]		120 [-0.0051]	
	530	0.22	0.24	2.7 [-0.13]	2.6 [-0.061]	170 [-0.0057]	90 [-0.0018]
	540	0.21	0.22	2.4 [-0.18]	2.4 [-0.096]	260 [-0.0053]	150 [-0.0025]
	550	0.21	0.18	2.2 [-0.21]	2.2 [-0.19]	430 [-0.0047]	270 [-0.0022]
	560	0.16		2.0 [-0.22]		600 [-0.0023]	250 [-0.0010]
371	520	0.28		2.3 [-0.066]		70 [-0.0040]	
	530	0.27	0.31	2.2 [-0.12]	2.2 [-0.066]	150 [-0.0042]	90 [-0.0017]
	540	0.22	0.27	2.1 [-0.16]	2.1 [-0.11]	300 [-0.0038]	160 [-0.0025]
	550	0.20	0.20	1.9 [-0.17]	2.0 [-0.14]	500 [-0.0032]	220 [-0.0025]
	560	0.18	0.20	1.6 [-0.19]	1.8 [-0.16]	800 [-0.0017]	270 [-0.0018]
290	520	0.34		2.0 [-0.054]		70 [-0.0034]	
	530	0.30		1.9 [-0.13]		110 [-0.0046]	
	540	0.24	0.29	1.9 [-0.17]	1.9 [-0.10]	190 [-0.0043]	110 [-0.0018]
	550	0.25	0.23	1.7 [-0.21]	1.8 [-0.13]	290 [-0.0039]	100 [-0.0017]
	560	0.24	0.20	1.5 [-0.24]	1.6 [-0.14]	500 [-0.0032]	690 [-0.00099]
230	520	0.37		1.9 [-0.066]		70 [-0.0031]	
	530	0.30	0.34	1.8 [-0.12]	1.7 [-0.070]	90 [-0.0037]	50 [-0.0092]
	540	0.24	0.30	1.8 [-0.17]	1.6 [-0.11]	200 [-0.0032]	70 [-0.0014]
	550	0.25	0.21	1.6 [-0.21]	1.6 [-0.13]	270 [-0.0032]	180 [-0.0016]
	560	0.17	0.22	1.4 [-0.21]	1.4 [-0.16]	680 [-0.0023]	200 [-0.0010]
121	520	0.49		1.2 [-0.087]		60 [-0.0018]	
	530	0.35	0.47	1.3 [-0.12]	1.2 [-0.069]	70 [-0.0020]	70 [-0.00048]
	540	0.26	0.3	1.2 [-0.13]	1.2 [-0.090]	120 [-0.0017]	330 [-0.00082]
	550	0.17	0.25	1.2 [-0.12]	1.2 [-0.11]	160 [-0.0011]	180 [-0.0011]
	560	0.15	0.16	1.0 [-0.12]	1.1 [-0.11]	380 [-0.00052]	40 [-0.00053]

Table S4. Fitting results (Eq S2) of **1p** and **2p** at the probe wavelengths corresponding to the GSB of LR_Q (670 nm) for different values of pump fluence. The error is estimated in the order of 10% from repeated measurements. Fitting amplitudes are reported between square brackets.

LR_Q (670nm)						
Pump fl. ($\mu J/cm^2$)	τ_{e-e}		τ_{e-ph} [A_1]		τ_{plex} [A_2]	
	**1p**	**2p**	**1p**	**2p**	**1p**	**2p**
514	0.34	0.30	2.9 [0.036]	2.8 [0.015]	510 [-0.0023]	710 [-0.0048]
371	0.29	0.32	2.4 [0.032]	2.0 [0.033]	520 [-0.0017]	710 [-0.0043]
290	0.20		2.1 [0.038]	2.0	390 [-0.0024]	490 [-0.0042]
230	0.64		1.7 [0.065]	1.5	130 [-0.00065]	320 [-0.0036]
121	0.77		1.2 [0.061]	1.6	150 [-0.0014]	360 [-0.0024]

References

(1) Xia, H.; Bai, S.; Hartmann, J.; Wang, D. Synthesis of Monodisperse Quasi-Spherical Gold Nanoparticles in Water via Silver(I)-Assisted Citrate Reduction. *Langmuir* **2010**, *26*, 3585–3589.

(2) Bonomi, R.; Cazzolaro, A.; Prins, L. J. Assessment of the Morphology of Mixed SAMs on Au Nanoparticles Using a Fluorescent Probe. *Chem. Commun.* **2011**, *47*, 445–447.

(3) Peruffo, N.; Gil, G.; Corni, S.; Mancin, F.; Collini, E. Selective Switching of Multiple Plexcitons in Colloidal Materials: Directing the Energy Flow at the Nanoscale. *Nanoscale* **2021**, *13*, 6005–6015.

(4) Del Fatti, N.; Voisin, C.; Achermann, M.; Tzortzakis, S.; Christofilos, D.; Vallée, F. Nonequilibrium Electron Dynamics in Noble Metals. *Phys. Rev. B - Condens. Matter Mater. Phys.* **2000**, *61*, 16956–16966.
